# Silver ions promote blebs growth in U251 glioblastoma cell by activating nonselective cationic currents

**DOI:** 10.1038/s41598-019-49198-7

**Published:** 2019-09-09

**Authors:** Francesco Ragonese, Lorenzo Monarca, Federica Bastioli, Cataldo Arcuri, Loretta Mancinelli, Bernard Fioretti

**Affiliations:** 1 Department of Chemistry, Biology and Biotechnologies, Via Elce di Sotto 8, University of Perugia, Perugia, Italy; 2 Department of Experimental Medicine, Piazzale Gambuli 1, University of Perugia, Perugia, Italy

**Keywords:** Permeation and transport, CNS cancer

## Abstract

Glioblastoma (GBM) is the most common and aggressive human brain cancer with low prognosis and therefore the discovery of new anticancer agents is needful. Sulfydryl reagents, such as silver, have been shown to induce membrane vesiculation in several cellular models through a mechanism that has not been yet completely clarified. Using U251 glioblastoma cells, we observed that silver induced irreversible bleb formation of the plasma membrane. This morphological event was anticipated by an increase of intracellular Ca^2+^ associated to extracellular Ca^2+^ influx. Accordingly, using patch-clamp whole cell recording during silver ion application, inward current/s (IAg) at −90 mV were detected and cells were permeable to Ca^2+^ and monovalent ions such as Na^+^. IAg activation and the intracellular Ca^2+^ increase promoted by silver ions (Ag^+^) were prevented by co-application of 20 µM cysteine and 300 µM DIDS (4,4′-Diisothiocyanatostilbene-2,2′-disulfonic acid), suggesting a critical role of thiol groups in the biological effects of silver ions. IAg was partially inhibited by 1 mM Gd^3+^, an unspecific inhibitor of cationic currents. Cysteine, Gd^3+^ and extracellular free Ca^2+^ solution completely abolished blebbing formation promoted by Ag^+^. Furthermore, extracellular Na^+^ ion replacement with TEA or an increase of extracellular tonicity by sucrose (100 mM) reduced both size and growth of membrane blebbing. Our data suggest that Ag^+^ promotes the formation necrotic blebs as consequence of the increase of intracellular Ca^2+^ and intracellular hydrostatic pressure associated to the activation of cationic currents. Since silver-induced blebs were less evident in benign glial human Müller MIO-M1 cells, silver compounds could represent new adjuvant to anticancer agents to improve GBM therapies.

## Introduction

Glioblastoma is the most common and aggressive type of human brain tumor. Its poor prognosis largely derives from its diffused invasiveness into normal brain tissues which precludes successful surgical resection^1,2^. Glioblastoma cells can migrate through different ways based on various interactions between cells and extracellular matrix. Three-dimensional or amoeboid glioblastoma migration was observed when the cells are forced to pass through tight spaces. In particular, cells change their shape and volume following a highly regulated process such as membrane blebbing^3^. The formation of blebs protrusions promotes amoeboid migration that typically occurs around the vessels^4^. Notably, blebbing based motility is essential for metastatic cells to escape anti-tumor treatments^5^. Several ion channels and transporters have been reported to display a role in the malignancy of glioblastoma cells by modulating morphological changes. Transporters and ion channels can regulate these mechanisms through two different ways: by changing the concentration of internal Ca^2+^ and by modifying the volume of the cell, a process closely related to a two-dimensional migration^6^. Recently, it has been reported that intracellular Ca^2+^ agonist bradykinin promotes blebs formation and invasion in glioblastoma cells^7^.

Membrane blebbing is an outward budding process where a plasma membrane protrusion is transitory formed in timescale of tens of seconds^8^. The temporary behaviour of the bleb is associated to a transient increase of hydrostatic pressure in the cytoplasm close to plasma membrane as a consequence of acto-myosin cortex contraction^9^. The dissipation of hydrostatic pressure in the elastic network is delayed by the biophysical properties of the cytoplasm (poroelastic model). In the presence of defects of the plasma membrane, the transitory pressure increase causes a cytosol flow and bleb protrusion, caused by the interaction with the underlying cytoskeleton. During this phase, actin undetectably on bleb surface only becomes evident when cytosol inflation is slow. The membrane blebbing retraction is a consequence of the new formation of the myosin contraction machinery on the bleb inner surface^9^. Depending on the nature of the inducing agent, its concentration or cell line model, blebbing can become not transient, but can continuously grow until it produces extracellular vesicles^10^. For example, low concentrations of hydrogen peroxide promote transitory blebs whereas high concentrations promote a continuous blebs growth^11^. Bleb induction has been associated to membrane protrusion during apoptosis whereas continuous blebs growth can lead to cell damage and necrosis^11^. The result of a continuous bleb growth is the release of membrane vesicles, in the culture medium of cells from different tissues^10,12^. Same indications support the idea that both the blebs (transient and continuous) start from a common mechanism, but differ in the hydrostatic intracellular pressure profile^11^.

Sulfhydryl reagents represent a class of compounds able to induce blebbing and vesicles release^13^. Depending on the nature of the compound and the cell type, various cellular blebbing morphological responses have been described (acentric, symmetric and scallop blebs). The involvement of thiolic groups in the sulfhydryl reagents inducing vesicle release was demonstrated by studying thiols protective agents such as cysteine and glutathione. It has been suggested that the formation of blebs is related to defects in the functionality of specific thiolic (SH) groups having a fundamental role in controlling the plasma membrane permeability and the actin-myosin interaction^10,13^. Silver ion (Ag^+^) is among the sulfhydryl reagents able to induce blebbing. The present work addresses blebs formation in glioblastoma U251 cells and the alteration of plasma membrane permeability following Ag^+^ exposure. Herein, we studied the properties of ionic currents associated to the treatment with silver ions and the involvement of the intracellular Ca^2+^ homeostasis.

## Materials and Methods

### Cell culture

The human glioblastoma multiform cell line U251 (Cell Lines Service, GmbH), human Müller cell line MIO-M1 and epithelioid cervix carcinoma HeLa cell line were grown in Dulbecco’s Modified Eagle Medium (DMEM) supplemented with 10% heat-inactivated fetal bovine serum, 100 IU/ml penicillin/streptomicyn, and 200 mM of L-Glutammine. The flasks were incubated at 37 °C in a 5% CO_2_-humidified atmosphere. For all experiments U251 cells were plated at 15,000/ml in a 35 mm petri dish and used on day 3 of culture.

### Cytosolic Ca^2+^ measurements

Accordingly with our previous work^14^, cells were incubated with FURA-2-AM (3 μM; Sigma-Aldrich) for 45 min and extensively washed with external Ringer’s solution of the following composition (in mM): NaCl 140, KCl 5, CaCl_2_ 2, MgCl_2_ 2, MOPS 5, glucose 10, at pH 7.4. Cells were continuously perfused using a gravity-driven perfusion system, focally oriented onto the field of interest. The estimation of intracellular free Ca^2+^ concentration, was reported as change of the ratio between fluorescence emission at 510 nm obtained with 340 and 380-nm excitation wavelengths (optical filters and dichroic beam splitter were from Lambda DG4, Shutter Instruments). Ratiometric data were acquired every 3 sec and fluorescence determinations were performed using fluorescence microscopy system Zeiss (Axiozoom V16 and Axiocam 502 mono). The acquisition and analysis were driven by ZEN 2 software (Zeiss). Intracellular calibration of FURA-2 to obtain exact free intracellular Ca^2+^ concentration was carried out at the end of the experiments determining maximum and minimum 340/380 ratio of FURA-2 in the field of cells being studied. The maximum and minimum ratio was obtained with ionomicyn co-applicated with Ca^2+^ extracellular solution and with Ca^2+^ free plus EGTA respectively as described in O’Connor and Silver^15^. The Ca^2+^ concentration was calculated by using the relationship described for FURA-2 by Grynkiewicz *et al*.^16^. In some experiments the FURA-2 signal was expressed as % change of the 340/380 ratio, normalized to maximal signal obtained following 3 µM ionomycin application.

### Electrophysiology

Accordingly with our previous work^17^, whole-cell perforated patch clamp configuration was used for electrophysiological recordings from U251 cells. Currents and voltages were amplified with a HEKA EPC-10 amplifier, and analyzed with the PatchMaster and Origin 4.1 softwares. For online data collection, currents were filtered at 3 kHz, and sampled at 100 µsec/point. Membrane capacitance measurements were made by using the transient compensation protocol of PatchMaster. The external solution contained (in mM): NaCl 106.5, KCl 5, CaCl_2_ 2, MgCl_2_ 2, MOPS 5, glucose 20, Na-gluconate 30, (pH 7.25). Octanol (1 mM) + TRAM-34 (3 µM) and TEA (3 mM) were added to the external bathing solution to block gap-junctions and intermediate and Big conductance Ca^2+^-activated potassium channels^18^. The absence of Ca^2+^-activated K channel activation following Ag^+^ application was assessed by monitoring outward currents at 0 mV (Vh = 0) according to our previous reports^19^. The pipette solution contained (in mM) 57.5 K_2_SO_4_, 55 KCl, 5 MgCl_2_, 10 MOPS (pH 7.20). Amphotericin B (200 µM) was added to the pipette solution to achieve electrical access to the cytoplasm^18^ ranging between 10 and 20 MOhm within 10 min after seal formation. Electrophysiological recording was performed before visible bleb formation was observed. This allowed to carry out experiments without the heavy leakeage currents associated to increased membrane permeabilization.

### Blebbing images analysis

U251 cells were washed twice with PBS and treated with 3 μM of AgNO_3_ in Ringer’s solution. Pictures of cells were taken every 30 seconds at 20× magnification using an Axio Examiner (Zeiss) with CCD Axiocam 502 mono digital camera (Zeiss) for at least 30 minutes. Blebs number and area were measured using Zen 2 image processing software.

### Reagents and solutions

All analytical grade chemicals were used. Amphotericin B, Dimethyl sulfoxide (DMSO), L-Cysteine, Gadolinium (Gd^3+^) and Tetraethylammonium (TEA) were from Sigma Chemical Co (St. Louis, MO). DIDS and DCPIB were purchased from Tocris Bioscience. TRAM-34 was a kind gift of Dr. Heike Wulff. TRAM-34 and Amphotericin B were prepared as stock solutions in DMSO at 20 mM and 50 mM respectively whereas DIDS was prepared in carbonate buffer at 100 mM. The maximal DMSO concentration used was 0.1% v/v for recording solutions. Solutions of AgNO_3_ (Sigma-Aldrich) were prepared immediately before application. Solutions were applied by a gravity-fed superfusion system at a flow rate of 2 ml/min. Experiments were carried out at room temperature (18–22 °C). Data are presented as mean ± SE.

## Results

### Silver promotes Ca^2+^ influx

**Figure 1:**
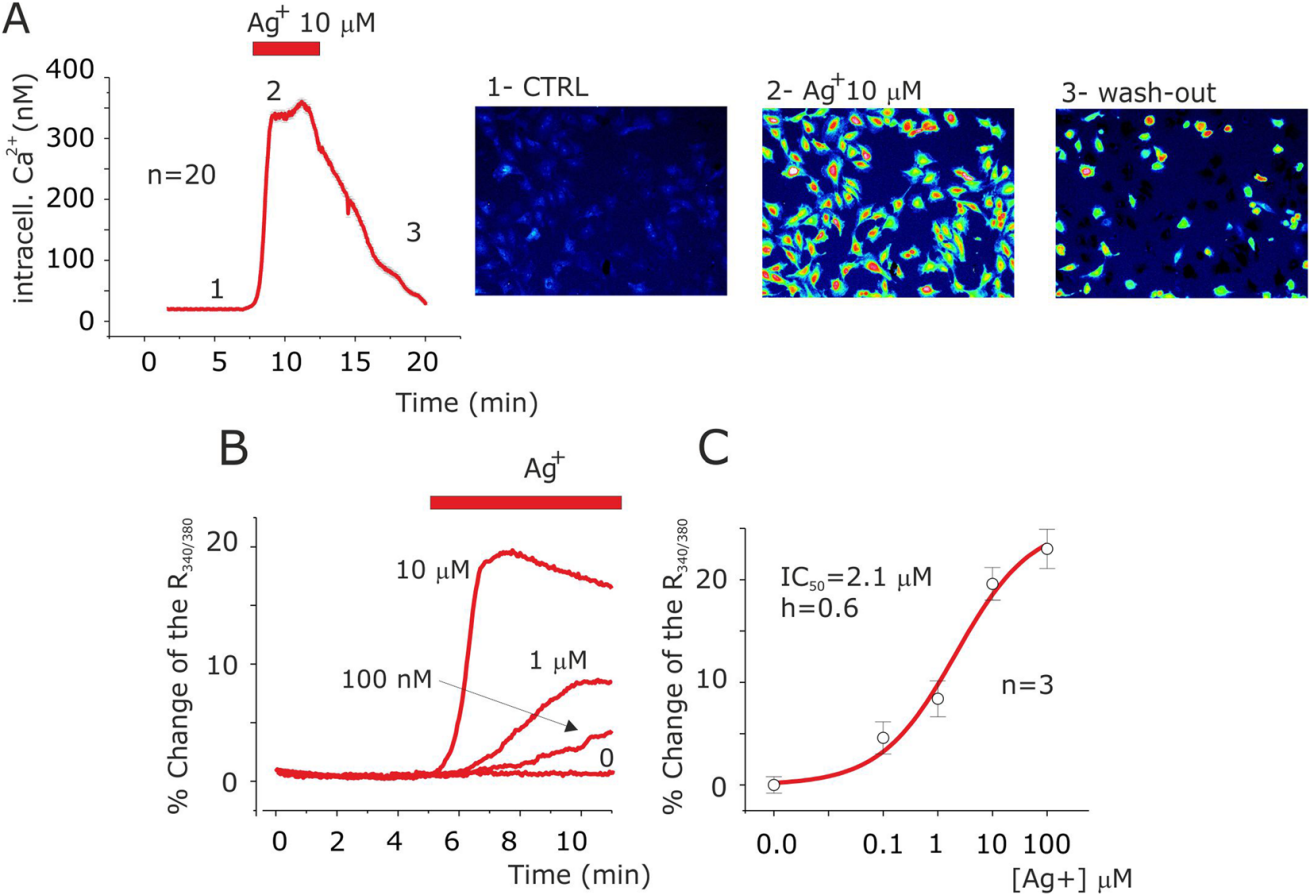
Ag^+^ increase intracellular Ca^2+^ levels in U251 glioblastoma cells. (**A**) Time course of mean intracellular Ca^2+^ concentration values, obtained every 3 s, by ratiometric living cellular fluorescent imaging of FURA-2 from 20 cells during application of 10 µM silver (grey bar). (**B**) Representative time course of intracellular Ca^2+^ levels at different silver concentrations. Note the effects of silver ions concentration on peak value and onset of fluorescence associated to ratio 340/380 emission of FURA-2. (**C**) Silver ions concentration and intracellular Ca^2+^ levels response curve relationship. The solid lines represents the best fit of the data points according to Hill’s equation Ix/Imax = 1/(1 + (IC_50_/[Ag^+^])^h^) where IC_50_ is half ratio 340/380 change in percentage and h is the Hill’s number. Bottom: pseudo-colour images of ratio 340/380 emission in CTRL (1), during 10 µM Ag^+^ application (2) and following washing-out (3) as indicated in the time course of panel A.

Firstly, we checked the effects of Ag^+^ application on intracellular Ca^2+^ level in U251 glioblastoma cells. Using FURA-2 Ca^2+^ imaging assay, the resting [Ca^2+^]_i_ was found to be 29.8 ± 0.5 nM; after 10 µM silver application it reached a plateau at 335 ± 2.2 nM with a mean time peak of 123 ± 2 s (n = 20) (Fig. 1A). All the cells were responsive to 10 µM of silver ion application and after washout the intracellular Ca^2+^ levels dropped to resting levels (Fig. 1A). Bath application of 10 µM NaNO_3_ did not modify the intracellular Ca^2+^ level (data not shown). The intracellular Ca^2+^ increase was dose dependent with EC_50_ of 2.1 µM (Fig. 1B,C) and also the peak time was concentration dependent with 323 and 402 s at 1 µM and 100 nM of Ag^+^ application respectively (Fig. 1B). The increase of intracellular calcium was observed in several human cell lines such as HeLa and MIO-M1, indicating that it is a common cellular process in response to Ag^+^ (Supplementary Fig. S1).

**Figure 2:**
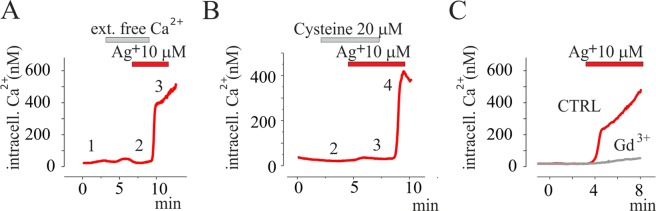
Silver promotes Ca^2+^ influx in a cysteine sensible manner. (**A**) Time course of mean intracellular Ca^2+^ concentration values, obtained every 3 s, via ratiometric living cells fluorescent imaging of FURA-2 from 20 cells in CTRL (1), during 10 µM silver application in extracellular Ca^2+^ free solution (2) and after addition of 2 mM Ca^2+^ in the presence of silver (2). (**B**) Time course of mean intracellular Ca^2+^ concentration values, obtained every 3 s, via ratiometric living cells fluorescent imaging of FURA-2 from 20 cells after 10 µM silver ions application in absence (CTRL, red line) and in presence of 1 mM of Gd^3+^ (grey line). (**C**) Time course of mean intracellular Ca^2+^ concentration values, obtained every 3 s, via ratiometric live cell fluorescent imaging of FURA-2 from 20 cells during application of 20 µM cysteine before (2) and after silver application (3). Note: the intracellular calcium increases following wash-out of cysteine in presence of Ag^+^ (4).

The slow time of Ca^2+^ mobilization promoted by Ag^+^ (in the minutes scale) suggests the occurrence of an extracellular Ca^2+^ influx. In accordance with this observation, the removal of extracellular Ca^2+^ ions prevented the silver-induced intracellular Ca^2+^ increase (Fig. 2A and Supplementary Fig. S3). In mast cells, Ag^+^-induced Ca^2+^ influx was independent from Store Operated Calcium Entry (SOCE), L-type Ca^2+^ currents, but involved a thiol-sensitive store-independent Ca^2+^ influx^20^. On the base of these observations, we tested the effects exerted on silver-induced Ca^2+^ increase by the incubation of the cells with: (i) the aspecific SOCE inhibitor Gd^3+^^21^, (ii) the L type voltage dependent Ca^2+^ currents antagonist nitrendipine^22^, (iii) the thiol reducing reagent cysteine^23^. Co-application of Ag^+^ with Gd^3+^ (30 and 300 µM) or nitrendipine (30 µM) did not abolish silver-induced Ca^2+^ increase (data not shown), whereas 1 mM Gd^3+^ preserved silver-induced Ca^2+^ increase (Fig. 2B). Moreover, silver co-application with cysteine 20 µM totally abolished the effect of silver that immediately recovered in a cysteine free solution (Fig. 2C and Supplementary Fig. S3).

### Silver activates a specific cation current (IAg) Ca^2+^ permeable

**Figure 3:**
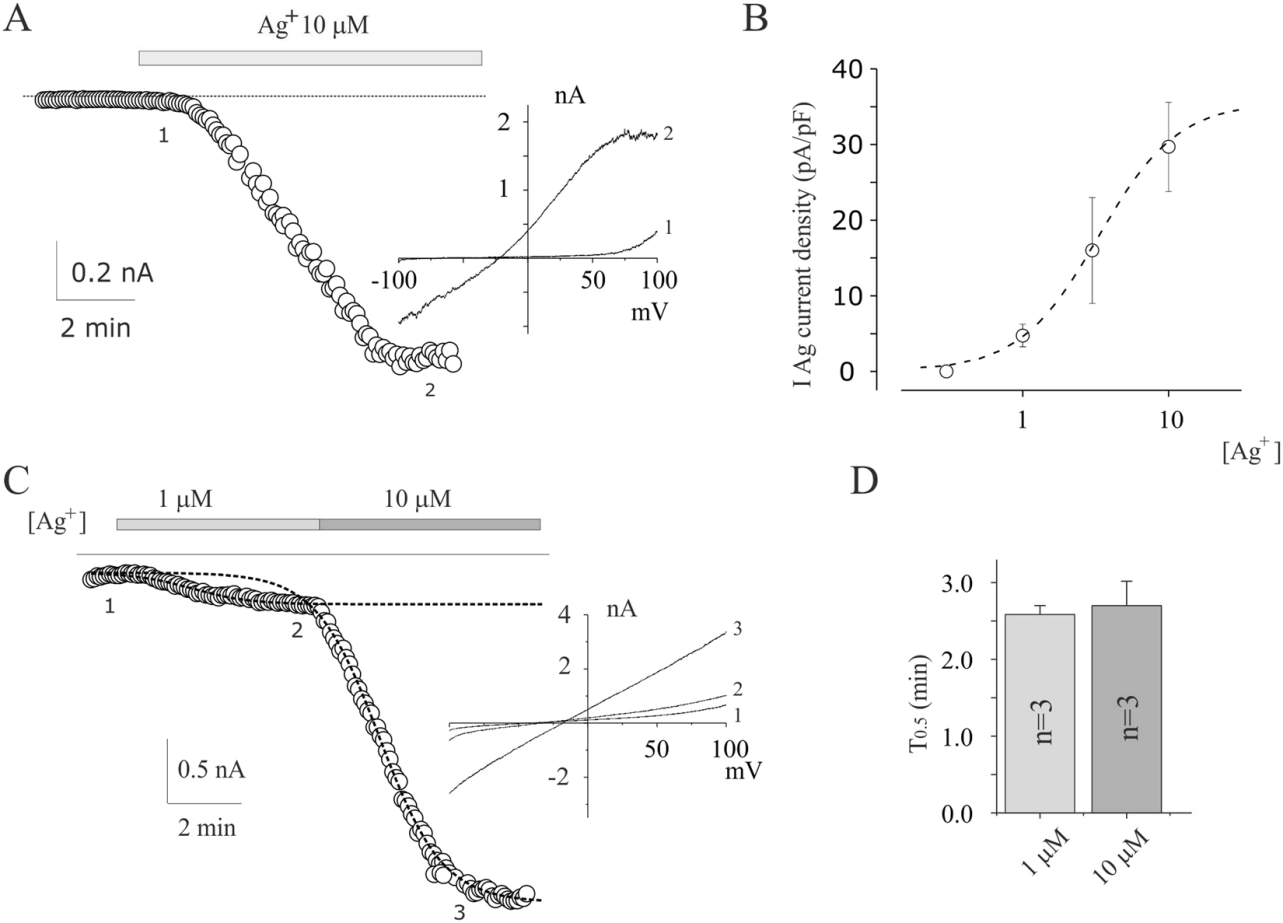
Silver-activated current in U251 glioblastoma cells. (**A**) Time course of inward current at −90 mV before and after application 10 µM Ag^+^. The data points represent the currents recorded at −90 mv during voltage ramp from −100 to 100 mv (1 second duration) from a Vh of 0 mV repeated every 5 s. Inset: representative current ramps taken at the times indicated in the relative time course. (**B**) Dose-dependent relationship of silver-activated currents at various silver ions concentrations. The dashed line represents the best fit of the data points according to Hill’s equation IAg(x) = IAg(max)/(1 + (IAg^50^/[Ag^+^])^h^), where IAg^50^ is the [Ag^+^] activating half Imax, and h is the Hill’s number. IAg(x) represents the IAg activated by a given [Ag^+^], and IAg(max) represents the maximal current density (i.e., at saturating [Ag^+^]). (**C**) Silver activated currents at different silver concentrations. The dashed line represents the best fit of the data points according to modified Boltzmann’s equation I(t) = Iss-I_0_/1 + exp (-(t-t_0_-T_0.5_)/slope), where Iss, T_0.5_ and I_0_ are the inward currents at the maximal current at each specified concentration, the half of time activation and inward currents at the beginning of silver application respectively^25^. Inset: representative current ramps taken at the times (1 and 2) indicated in the relative time course. (**D**) Bar blot of the mean time of activation (T_0.5_) estimated as described in C for 1 and 10 µM of Ag^+^ concentration.

Electrophysiological experiments were performed to further investigate the ionic mechanism of the effects of Ag^+^. To minimize the contribution of intermediate (KCa3.1) and Big (KCa1.1) calcium activated potassium currents, we performed electrophysiological recording in presence of the inhibitors TRAM-34 and TEA^18^ at the membrane voltage near the equilibrium potential of K^+^. In a physiological solution, in whole cell perforated patch-clamp configuration, 10 µM of Ag^+^ application activates an inward current at −90 mV (Fig. 3A) in all the U251 glioblastoma cells tested (n = 23), evaluated by applying voltage ramp protocols from −100 mV to +100 mV from a holding voltage (Vh) of 0 mV. We defined this current as silver-activated current (IAg). The mean IAg current density obtained from five experiments carried out in the same conditions, was −29.7 ± 5.9 pA/pF at −90 mV, and was partially reversible following wash-out (Supplementary Fig. S2) accordingly with the Ca^2+^ imaging experiments in Fig. 1A. The reversal potential was −21,4 ± 2,3 mV (n = 5) which excluded that IAg could represent Na^+^ or K^+^ selective currents, or leakage currents. In fact, the equilibrium potential of K^+^ and Na^+^ ions, calculated by Nernst’s equation, in our conditions was about −90 mV and +65 mV, respectively. Ag^+^ activated IAg current in a dose-dependent manner with IAg^50^, Hill’s coefficient and IAg(max) of 3.3 µM, 1.6 and 34.6 pA/pF, respectively (Fig. 3B). The IC_50_ of silver induced Ca^2+^ increase (Fig. 1B) and IAg^50^ current activation (Fig. 3B) were quite similar. In contrast to Ca^2+^ experiments, the temporal profile activation of IAg was silver concentration independent in a wide range of concentrations (2.6 ± 0.1 min and 2.7 ± 0.9 min at 1 and 10 µM of silver ions application, respectively n = 3, Fig. 3C e D).

**Figure 4:**
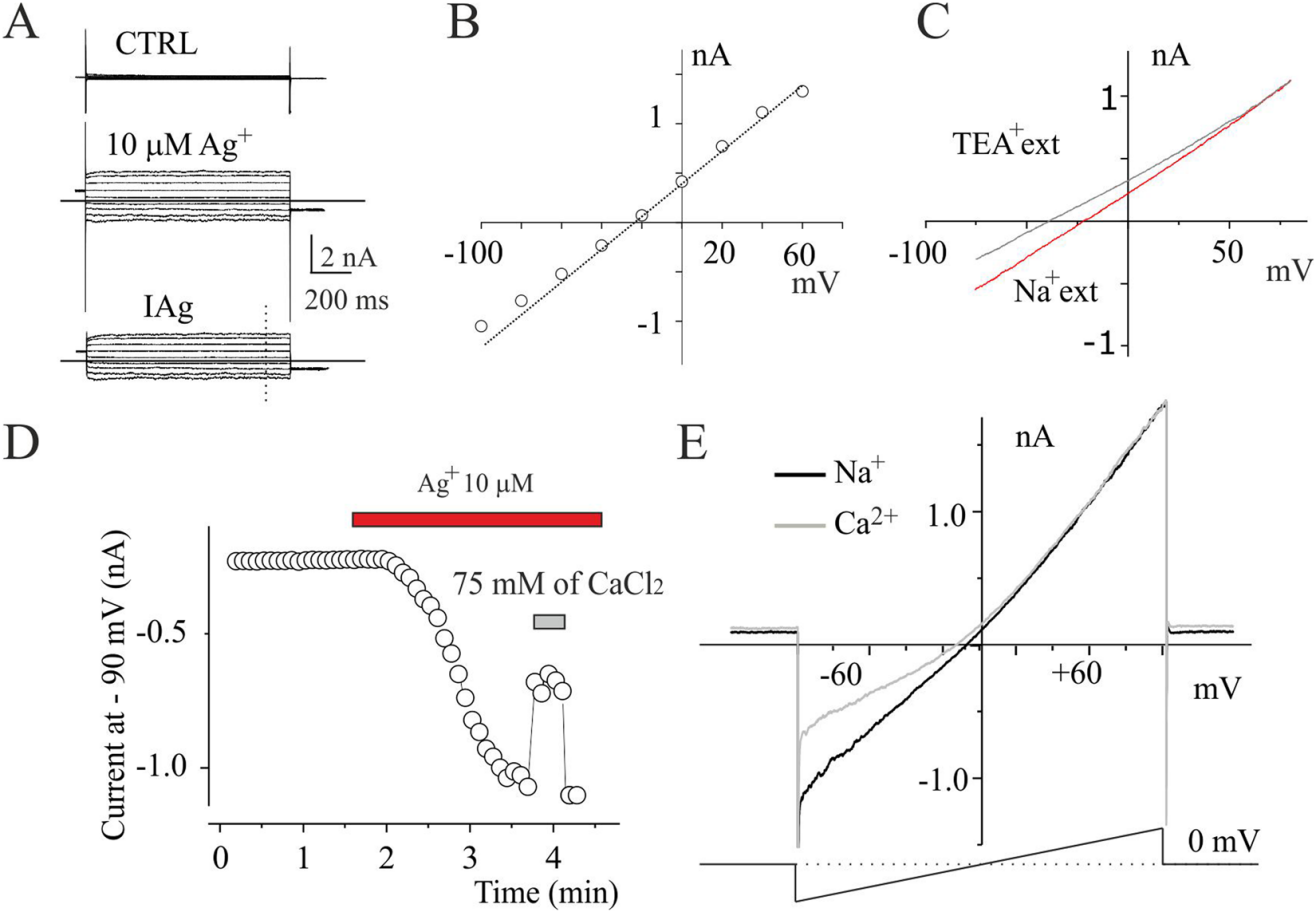
Permeability proprieties of silver-activated current in U251 cells. (**A**) Family of currents evoked by depolarizing steps from −100 to 60 mV, from V_holding_ of 0 mV, before (top) and after stable IAg activation obtained applying 10 µM of Ag^+^ (middle). Repolarization voltage at the end of each voltage step was −60 mV. The family of currents in bottom is the digital point-to-point subtraction between top and middle current family traces. (**B**) I-V relationship built with the current traces displayed in panel A (bottom) at the time indicated (dashed line). (**C**) I-V relationship of IAg obtained with ramp voltage step from −100 mV to 100 mV from a V_holding_ of 0 mV recording before and after ionic external substitution of all Na^+^ with TEA. (**D**) Time course of IAg currents activation following silver application at −90 mV and Na^+^ physiological external solution replacement with CaCl_2_ (grey bar). (**E**) I–V relationships from experiment described in D before and after Na^+^ replacement with Ca^2+^.

To understand the biophysical properties of IAg_,_ such as voltage dependence and permeability, we recorded the current responses from U251 cells by applying a 1 second duration pulses from −100 to 100 mV (V_holding_ of 0 mv) before (Fig. 4A, CTRL) and after stable IAg activation (Fig. 4A, 10 µM Ag^+^). I-V relationship was built by estimation of the currents obtained from digital subtraction between currents in Ag^+^ and CTRL conditions at the end of the voltage steps (Fig. 4A,B). The current displayed instantaneous behaviour with no activation or inactivation voltage process at negative voltage test, whereas at membrane potential over 60 mV it was possible to see a voltage activation component developed during the pulse depolarization. The I-V relationship built at the end of voltage membrane pulses showed an ohmic behaviour in the range from −40 to +40 whereas outside of this range a slight deviation from linearity was generally observed (Fig. 4B). Ionic substitution was used to investigate the permeability properties of IAg. Replacement of extracellular Na^+^ ions with Tetraethyl Ammonium (TEA) ions shifted the reversal potential of about 20 mV in negative direction (Fig. 4C), whereas the replacement of chloride anions with gluconate anions did not modify the I-V relationship (data not shown). The significant permeability to large cations as TEA suggests that IAg does not represent a leakage current through the plasma membrane, but a permeation process associated to typical ion channels^24^. In order to define the Ca^2+^ permeability of IAg current we studied the effects of extracellular solution replacement all sodium with 75 mM of CaCl_2_^25^. Inward current amplitudes were 50% reduced respect to control under these conditions (Fig. 4D,E), while outward currents were hardly affected (Fig. 4E). Together, these data suggest that Ca^2+^ acts as a permeant blocker as reported for other nonselective cation currents^26^, and indicate that the current activated by Ag^+^ is a cationic nonselective current permeable to Ca^2+^ ions.

**Figure 5:**
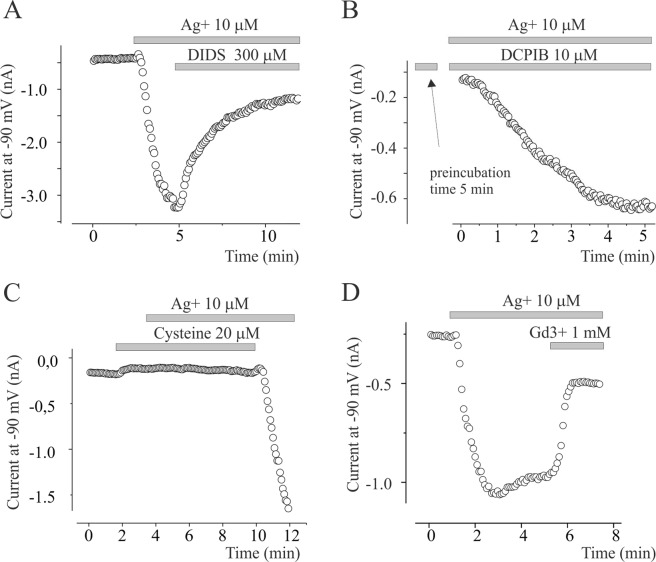
Pharmacological profile of IAg. (**A**) Time course of inward current at −90 mV of IAg current activation following 10 µM of Ag^+^ application and its block by 300 µM of DIDS. (**B**) IAg activation following acute Ag^+^ application in a cell pre-incubated for 5 minutes with 10 µM of swelling activated chloride current blocker DCPIB before Ag^+^ application. (**C**) Pre-incubation of 20 µM of cysteine prevented IAg activation by 10 µM ions application. Note the quickly activation of IAg following cysteine washout. In all dot plot, the single circle represents the current at −90 mV estimated from voltage ramp protocol applied every 5 s. The gray bars indicate the time of application of Ag^+^ and inhibitors. (**D**) Time course of inward current at −90 mV of IAg current activation following 10 µM of Ag^+^ application and its block by 1 mM of Gd^3+^.

The IAg share similar biophysical properties of the unselective Na^+^ permeable conductance previously reported in *Xenopus sp*. oocyte activated by silver^27^. Since the IAg expressed in Xenopus oocyte is inhibited by an aspecific chloride blocker such as 4,4′-Diisothiocyano-2,2′-stilbenedisulfonic acid (DIDS), we verified whether this could also occurred for our currents. 300 µM DIDS inhibits about 80% the silver-activated cationic currents (Fig. 5A). In contrast, selective inhibitor of swelling-activated chloride currents (IClsw), 4-(2-Butyl-6,7-dichloro-2-cyclopentyl-indan-1-on-5-yl oxobutyric acid (DCPIB) did not inhibit IAg in the same experimental condition (data not shown). Pre-incubation of DCPIB for 5 minutes did not modify the activation process induced by Ag^+^ (Fig. 5B). In contrast, DCPIB was more efficient to block IClsw expressed in U251 and activated by hypotonic solution (data not shown). This suggests that the effects of DIDS is not related to blocking action on IClsw. According to Schnizler *et al*.^27^, 20 µM cysteine prevented the activation of IAg, whereas 1 mM Gd^3^^+^ inhibited it (Fig. 5C,D).

Since IAg currents promoted Na^+^ influx, we evaluated the impact of silver application on the resting membrane potential. Using whole-cell perforated configuration in current-clamp mode, we measured the V_resting_ before and after 10 µM Ag^+^ application. Resting membrane potential was −48.6 ± 5.4 mV (n = 7) in control conditions, which dropped down to −1.7 ± 9.2 (n = 7) following application with Ag^+^ (data not shown, p < 0.05). Since the increase of the intracellular Na^+^ accumulation and membrane depolarization has been demonstrated to be involved in intracellular Ca^2+^ increase by promoting reverse mode of Na/Ca exchanger^28^, we verified its involvement of Na/Ca exchanger in silver-induced Ca^2+^ increase. Pre-incubation of the cells with the inhibitor of Na/Ca exchanger YM244769^28^, did not abolish the ability of Ag^+^ to increase intracellular Ca^2+^ (data not shown), thus suggesting that it was not dependent on reverse mode of Na/Ca exchanger.

### Silver promotes blebbing by increasing Na^+^ influx, intracellular Ca^2+^ concentration and hydrostatic pressure

**Figure 6:**
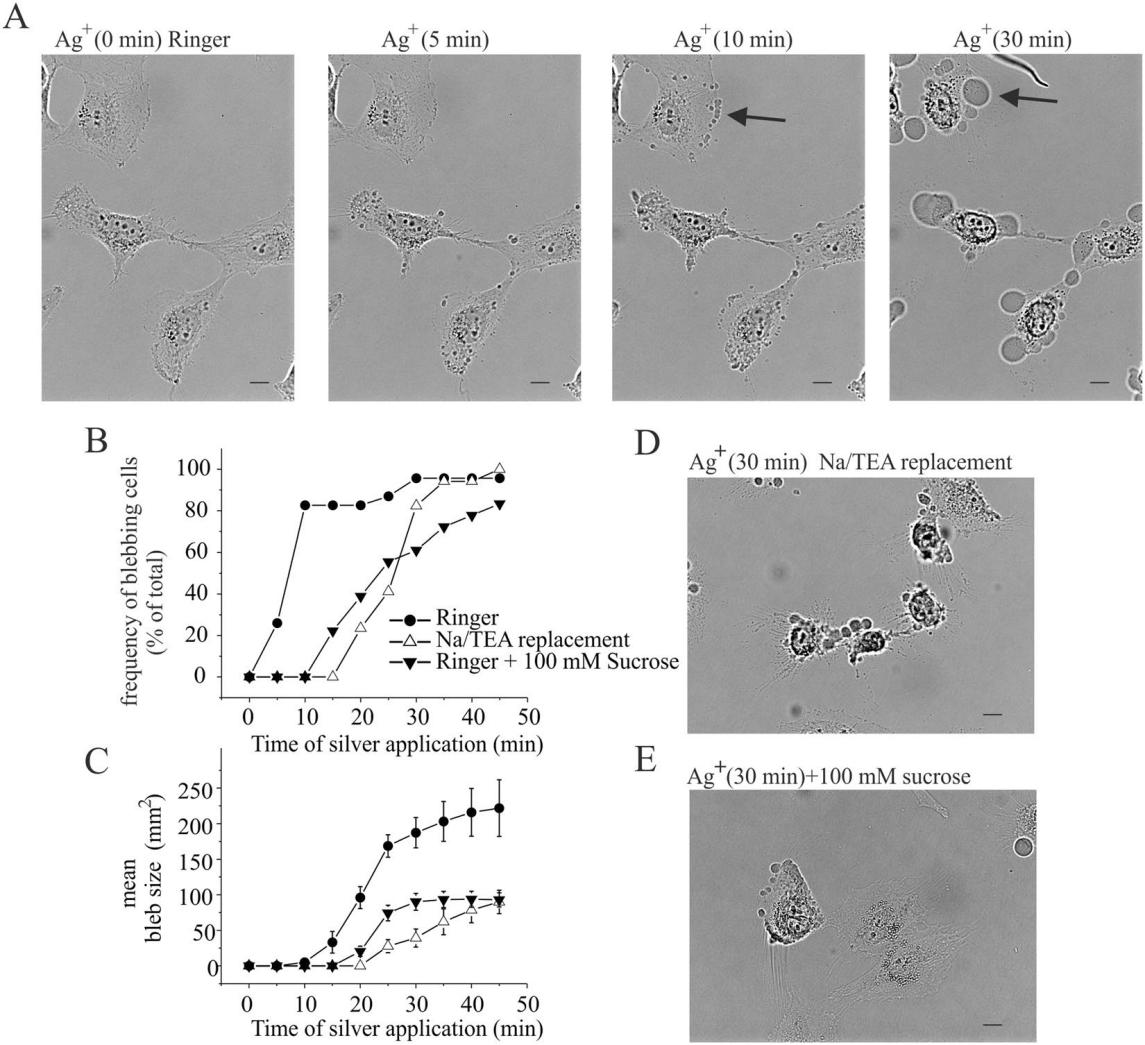
Silver induced membrane blebbing is dependent to IAg current activation. (**A**) Time-lapse imaging in bright light of field of U251 cells during the Ag^+^ treatment (3 µM) at 0, 5, 10 and 30 minutes after application. (**B**) Frequency of blebbing cells expressed as % of total cells analyzed during the time of Ag^+^ application (3 µM) in extracellular Ringer perfusion (circles), by substitution Na^+^ with TEA (empty triangles) and by increase extracellular osmolarity with 100 mM of sucrose (solid triangles). (**C**) Mean bleb size estimated as area of bleb after Ag^+^ application (3 µM) in extracellular Ringer solution perfusion (circles), by substitution Na^+^ with TEA (empty triangles) and by increase extracellular osmolarity with 100 mM of sucrose (filled triangles). (**D**) Representative image of U251 cell culture after 30 minutes of 3 µM of application in hypertonic condition (Ringer solution +100 mM of sucrose). (**E**) Representative image of U251 cell culture after 30 minutes of 3 µM of Ag^+^ application in Na^+^ replacement with TEA extracellular solution. Scale bar 20 µm.

After few minutes of Ag^+^ application, we detected the appearance of membrane blebs. As shown in Fig. 6A,B, during the treatment, the percentage of blebs positive cells increased and after 10 minutes, this process involved almost all the u251 analysed cells (Fig. 6A,B). No correlation between number of blebs/cell and cell size was observed. Positive cells generally displayed several blebs protrusion that tended to fuse together during time forming big blebs (see arrows in Fig. 6A, Supplementary Video S1). In some cases, single bleb grew in size without fusion. Single bleb size analysis obtained by unfused blebs was shown in Fig. 6C. Blebs grew during the time until they reached the size area of about 200 µm^2^ within 45 minutes (Fig. 6C, Supplementary Video S1). Perfusion with trypan blue dye marked the nuclei of blebbing cells indicating changes of membrane permeability typical of the necrotic process (Supplementary Fig. S4). No vesiculation was observed during the course of our experiments, but it is not possible to exclude that this process can occur in a later phase of the blebbing process^12^. Volume increase, related to blebs formation, could be due to an intracellular hydrostatic pressure increase driven by osmolites influx such as Na^+^. Since IAg is a Na^+^ influx, we investigated Na^+^ IAg-driven role as the main actor of hydrostatic pressure increase during blebs formation. For this purpose, we tested the Na^+^ substitution with less IAg-permeable cation TEA (Fig. 4C) in blebs formation. In this condition, blebs appearance was delayed (Fig. 6B,D) and their growth was reduced (Fig. 6C,D) as compared to control condition. To study the involvement of intracellular hydrostatic pressure in blebs formation, we performed experiments in hypertonic condition by adding sucrose (100 mM) to Ringer’s solution. The effects of hypertonic solution in blebs formation were similar to those observed in Na^+^ replacement with TEA (Fig. 6B–E). Notably, hypotonic stimulation alone, although increases cell volume as consequence of water influx, was not able to promote blebbing formation (data not shown).

These data demonstrate that Na^+^ and water influx play a role in silver promoted membrane blebbing, likely deriving from IAg currents activation. According to this interpretation, IAg blockers, such as cysteine, Gd^3+^ and DIDS, abrogated or reduced the effects of silver promoted morphological changes (Supplementary Fig. S5). Ca^2+^ role in silver induced blebbing was also investigated. In extracellular Ca^2+^ free medium silver was not able to promote membrane blebbing (Supplementary Fig. S5). Altogether, these data indicate that the increase of Ca^2+^ and Na^+^ intracellular concentration associated to IAg currents activation is the main event occurring in silver induced blebbing in U251 glioblastoma cells.

**Figure 7:**
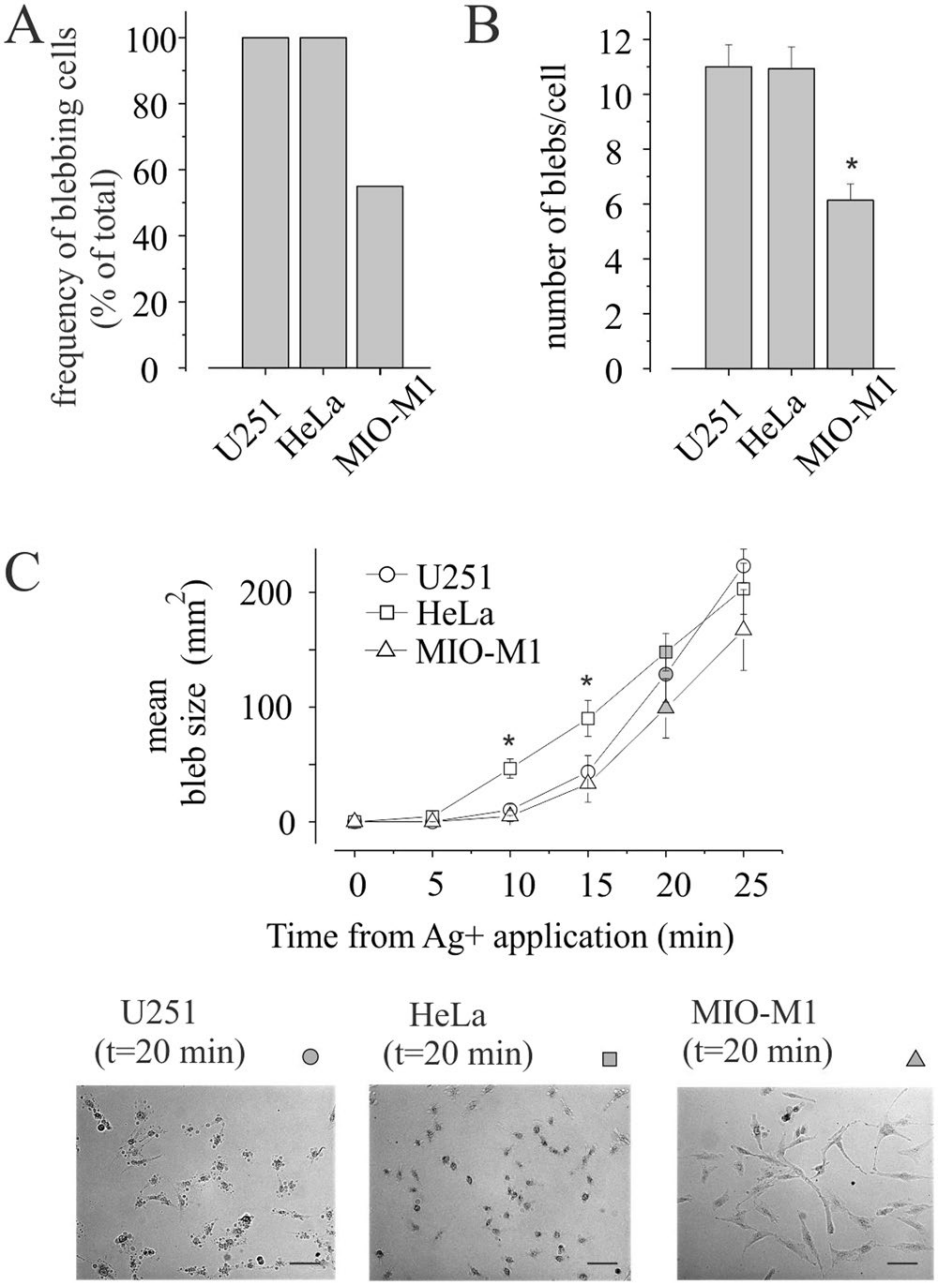
Normal human glial cells are less sensityve to silver induced membrane blebbing. (**A**) Percentage of blebs positive cells at 20 min of 3 µM Ag^+^ exposure in U251, Hela and MIO-M1 cell lines. (**B**) Number of blebs/cell estimated in 20 blebbing positive cells at 20 min of 3 µM Ag^+^ exposure in U251, Hela and MIO-M1 cell lines. (**C**) **Top:** Mean bleb size estimated as area of blebs measured every 5 minutes from Ag^+^ application (3 µM) on U251 (round) cell line, HeLa (square) and MIO-M1 (triangle). Note the major blebs size at 10 and 15 minutes of HeLa when compared to U251 and MIO-M1 (*p < 0.05). **Bottom:** Representative images of blebbing cell in U251, HeLa and MIO-M1 at 20 minutes of 3 µM Ag^+^ application as indicating in the graph. Scale bar 100 µM.

Blebs formation was observed after Ag^+^ application also in HeLa and MIO-M1 human cell lines (Fig. 7). Specifically, HeLa cells display blebs about 20 min after silver application with a number of blebs/cell ratio similar to U251 glioblastoma cells line (Fig. 7A,B). Blebs growth, estimated as blebs size, is instead higher as compared to U251 glioblastoma cell (assessed until 20 minutes after silver application; Fig. 7C). Interestingly, the human glial Müller MIO-M1 cell line displays, in the same condition of silver ions treatments, only a half of the blebbing cells, and with a significantly lower ratio of blebs/cell, with respect to both U251 and HeLa cell lines (Fig. 7B). Although the blebbing process is less evident in normal glial MIO-M1 cells, their growth was similar to that assessed in the U251 cell line (Fig. 7C).

## Discussion

In this paper, we studied the electrophysiological and morphological effects of Ag^+^ on human glioma multiforme U251 cells. Cell exposure to Ag^+^ in the micromolar range induced an irreversible growth of blebs protrusions. Cellular blebbing is a dynamic expansion of the plasma membrane following local disruption of its interaction with the actin submembrane cortex, accompanied by an increase of transient and local intracellular pressure. Depending on the nature of the stimulus or the cell type, blebs formation could be either transient and reversible, or irreversible and continuously growing^11^. The first one occurs in many cellular activities such as migration, adhesion and cytokinesis, while irreversible blebbing has been associated to the plasma membrane changes during programmed cell death^29^. In HeLa cells, the exposure to micromolar range of hydrogen peroxide concentration, promotes small and transient blebs associated to apoptosis. Rising to millimolar concentration, results in constant rate growth of bleb formations associated to necrotic process and inhibition of their retraction. Microscopic analysis displays a common starting mechanism for both bleb types (reversible and irreversible) with a similar diameter of the neck linking the membrane protrusion to the cytoplasm membrane^11^. In our experiments, cell exposure to micromolar silver concentration promotes blebs formation with a constant growth rate, a process similar to that associated with necrosis. According to this interpretation, application of trypan blue dye on blebbing cells marks cells nuclei due to the change of membrane permeability associated to the necrotic event^11^.

Apoptotic blebbing coincides with cell shrinkage whereas necrotic blebs are accompanied to Na^+^ influx driven by nonselective cation channels and cell swelling^30,31^ defined as Necrotic Volume Increase (NVI). Similarly, we found that silver ions cell treatment activates, in a concentration dependent manner, a nonselective cationic currents (IAg) permeable to Na^+^, K^+^ and Ca^2+^. The role of Na^+^ influx associated to IAg in bleb formation was demonstrated by using pharmacological and biophysical approaches. The IAg inhibitors, such as DIDS, Gd^3+^ and cysteine prevented blebs appearance, whereas the Na^+^ extracellular substitution with TEA or the increase of extracellular osmoalarity, reduced the blebs growth. These data demonstrate that the involvement of IAg currents in blebs formation may be associated to Na^+^ and water influx that increase the intracellular pressure. Surprisingly, we observed that the increase of intracellular hydrostatic pressure, obtained by applying hypotonic conditions, resulted in cell swelling without blebs formation. This suggests that, beside hydrostatic pressure, a further mechanism induced by silver ions is necessary to promote necrotic blebbing event. It is likely that it involves the Ca^2+^ signalling, since IAg Ca^2+^-permeable current and extracellular Ca^2+^ removal abolished the silver induced blebbing. In some cases, the intracellular Ca^2+^ increase was demonstrated to cause the local destabilization of the link of the plasma membrane with the actin in the submembrane cortex and to promote bleb formation^32,33^. Intracellular hydrostatic pressure is balanced by membrane tension dependent upon the cytoskeletal network under normal conditions. During bleb formation the reduction of local membrane tension caused by cytoskeletal destabilization, represents the point where cytosol flows into the bleb. The cytosol invasion according to Bernoulli’s principle is driven by the hydrostatic pressure gradient between the interior of the cell and the bleb^11^. Our proposed mechanism for silver-induced blebbing formation is based on IAg-driven Na^+^ and Ca^2+^ influxes responsible of hydrostatic pressure and Ca^2+^ concentration increase respectively. In particular, the intracellular Ca^2+^ increase is necessary for bleb induction, whereas Na^+^ influx is necessary to bleb growth.

Several electrophysiological effects of Ag^+^ have been reported in different cellular models. In *Helix pomatia* neurons, Ag^+^ application was described to increase intracellular Ca^2+^ concentration and to activate aspecific cationic currents^34^ but their molecular nature was not identified. Similarly, in *Xenopus oocytes* Ag^+^ application increases an aspecific cationic Na^+^ permeable current conductance that was prevented by chloride current blockers DIDS and SITS but not by NPPB and 9AC. More interesting, reagents that reduce disulfide bridge like dithiothreitol (DTT) and cysteine abolished inward cation currents activated by Ag^27^. The IAg currents reported here share similar pharmacological and biophysical proprieties, such as DIDS, Gd^3+^ and cysteine sensibility, as compared to those described in *Xenopus* oocytes. It is however possible that the IAg currents recorded in our experiments flow through TRP-like channels sensible to redox status. In fact, it has been demonstrated that a class of TRP Ca-permeable cation channels is subjected to modifications of protein sulfhydryl groups and that these are sensitive to changes in the redox status^35^. Since silver-induced Ca^2^^+^ increase and IAg activation are both quickly reversible on wash-out, the receptor/s for Ag^+^ may be localized on the external side of plasma membrane. However, we cannot exclude the presence of cytosolic receptors for Ag^+^ based on the observation that an active sodium dependent transport of Ag^+^ was reported^27,36^.

Ag^+^ has been shown to be able to promote cell death and could represent a potential anticancer agent. We observed that U251 glioblastoma cells display a greater susceptibility to blebbing as compared to normal glial Müller MIO-M1 cell line. Since blebs formation is associated to necrotic induction, these data suggest the possible use of sulfhydryl reagents such as silver ions as new anticancer agent to induce selective necrotic program in cancer cells versus normal cells. The ability of Ag^+^ to generate hydroxyl radicals has been suggested to represent the underlying mechanism in inducing cytotoxicity and apoptosis by silver nanoparticles^37^. In fact, their action may be related to the capability to enter cells and release Ag^+^^38^. Understanding the molecular nature of the IAg activation, as the triggering factor of the necrotic bleb formation in glioblastoma cell lines, could be useful in developing a silver-based new agent, as a promising strategy for anti-cancer therapy and to improve the knowledge about the emerging application of silver in nanomedicine.

## Supplementary information

Supplementary Figures

Supplementary Video S1
