# Supplementary material for: Silver ions promote blebs growth in U251 glioblastoma cell by activating nonselective cationic currents
Source: Sci Rep. 2019 Sep 9;9:12898. doi: 10.1038/s41598-019-49198-7 (PMC6733836; doi:10.1038/s41598-019-49198-7)
Supplement: Supplementary file 1 — Supplementary Figures [file 41598_2019_49198_MOESM1_ESM.pdf]

Silver ions promote blebs growth in U251 glioblastoma cell by activating nonselective cationic current.

Francesco Ragonese, Lorenzo Monarca, Federica Bastioli, Cataldo Arcuri, Loretta Mancinelli and Bernard Fioretti

SUPPLEMENTARY FIGURES

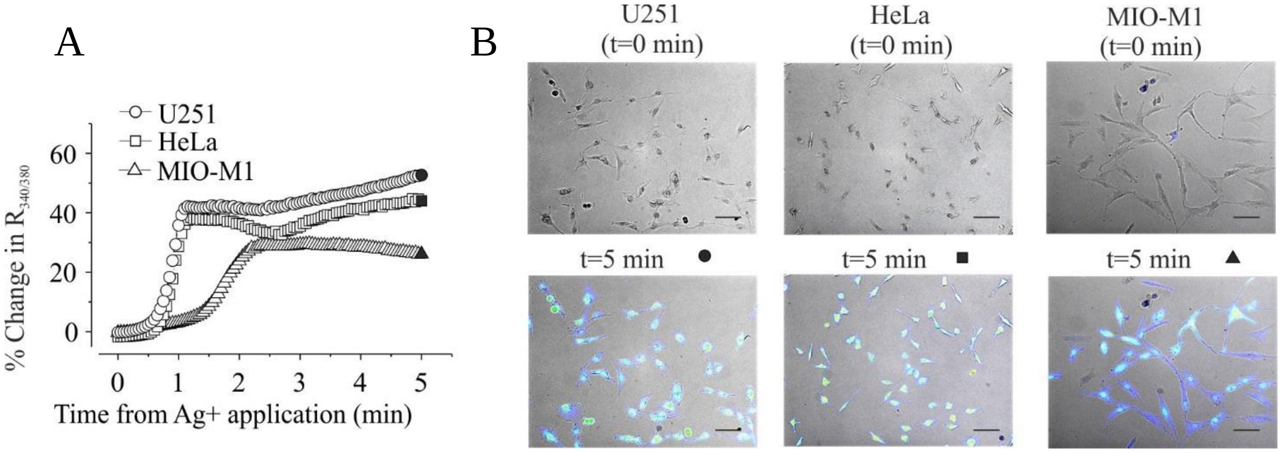

Supplementary Fig. S1. Silver ions increase intracellular Ca^{2+} levels in different human cell lines. A) Intracellular calcium levels evaluated by fluorescent imaging with FURA-2 from 20 cells during application of 3 µM of silver ions in cervical carcinoma HeLa (squares) [1] and immortalized human Müller cell line MIO-M1 (triangles) [2] compared with U251 glioblastoma cell lines (circles). **B)** Merge of brightfield and pseudo-colour of ratio 340/380 emission images of above mentioned cell lines before (0 min) and after 5 minutes application of 3 µM of Ag^+. Intracellular calcium levels is indicated on a rainbow scale with blue represent low $[Ca^{2+}]_i$ and orange/red high $[Ca^{2+}]_{i.}$. Scaling bars: 100 µm.

1. Gey G. O., Coffman W. D., Kubicek M. T., 1952. Tissue culture studies of the proliferative capacity of cervical carcinoma and normal epithelium. Cancer Res. 12: 264–265
2. Limb G. A., Salt T. E., Munro P. M., Moss S. E., Khaw P.T. In vitro characterization of aspontaneously immortalized human Müller cell line (MIO-M1). *Invest Ophthalmol Vis Sci*. **43(3)**, 864-9, (2002).

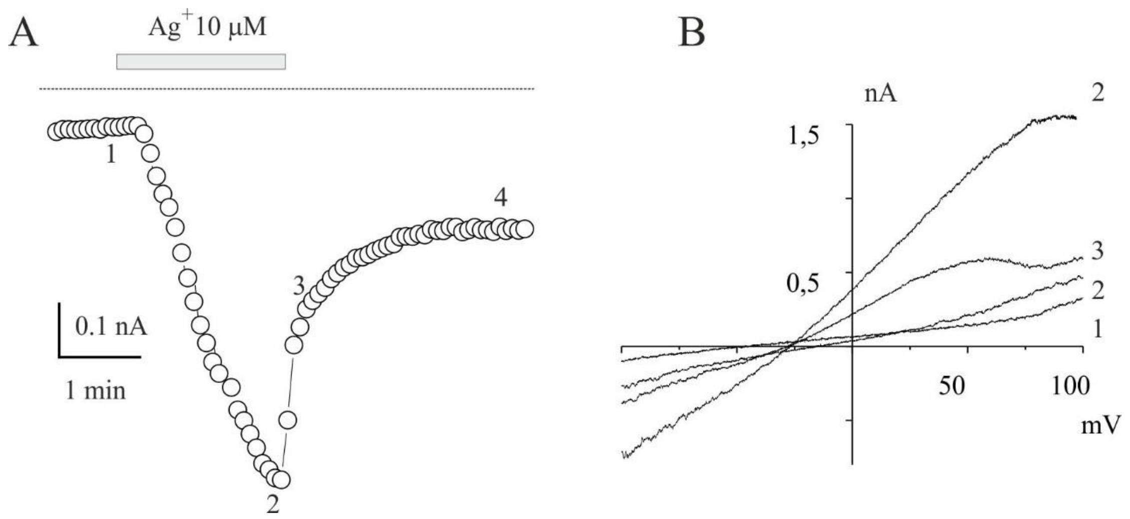

Supplementary Fig. S2. IAg was partially reverted after wash out. A) Time course of inward current at -90 mV before (1), after application 10 μM Ag^{+} (2) and during wash-out (3 and 4). The dates points represent the currents recorded at -90 mv obtained during voltage ramp from -100 to 100 mv (1 second duration) from a Vh of 0 mV repeated every 5 s. **B)** Representative current ramps taken at the times indicated in the relative time course shown in A.

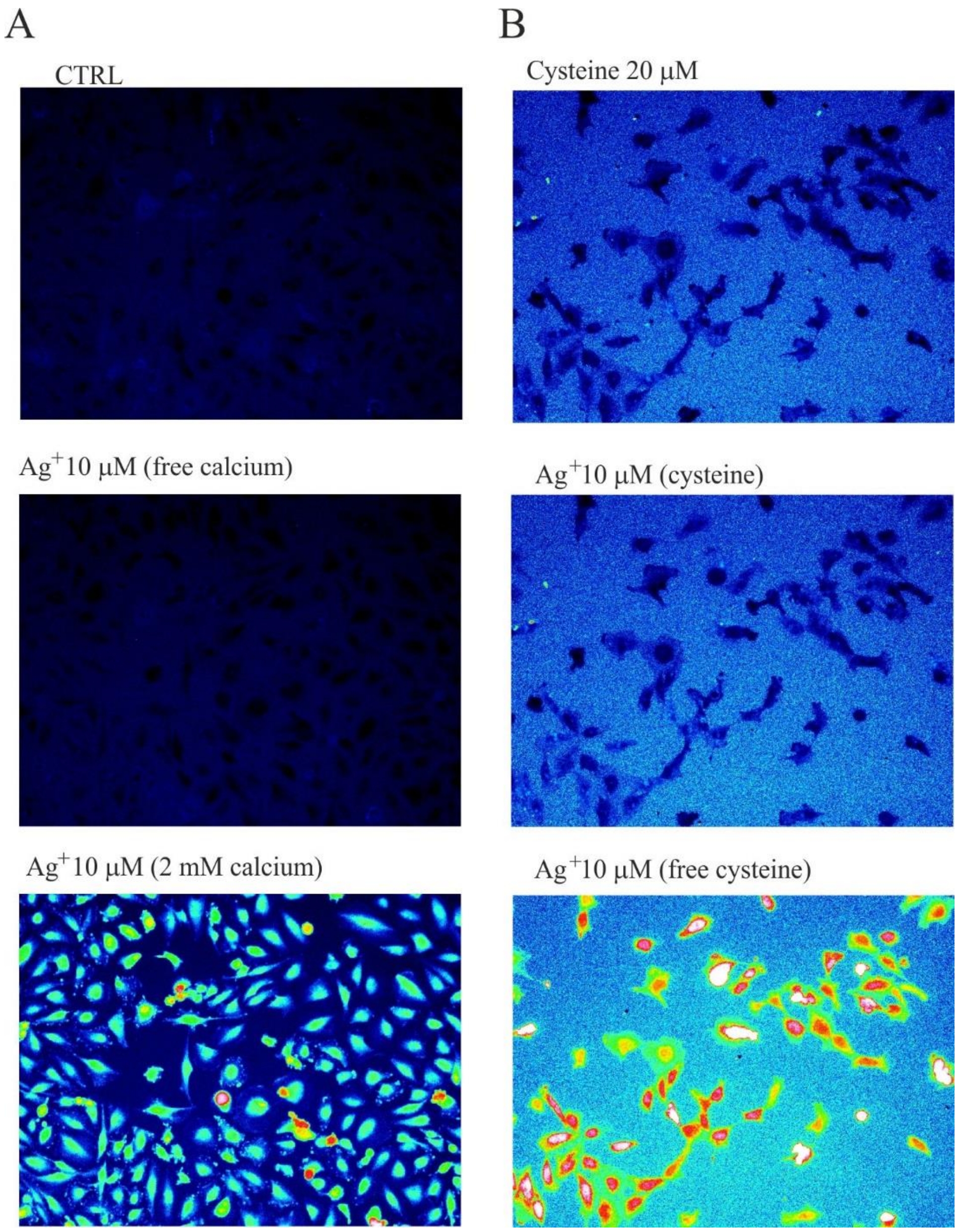

Supplementary Fig. S3. Representative pseudo-colours calcium imaging of experimental data from fig. 2. A) and B) Pseudo-colours calcium imaging captures at the time indicating in Fig 2A and 2B respectively. Intracellular calcium levels is indicated on a rainbow scale with blue represent low $[Ca^{2+}]_i$ and orange/red high $[Ca^{2+}]_{i.}$.

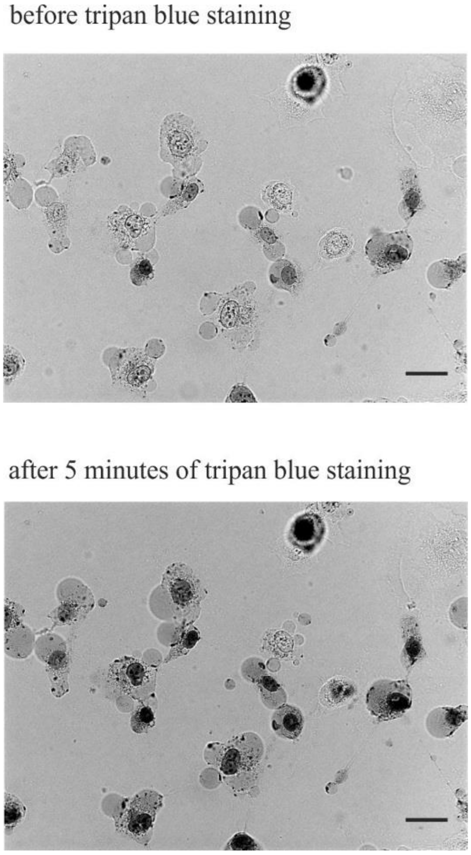

Supplementary Fig. S4. Tripan blue assay in Ag^{+} -induced blebbing cells. Images of U251 exposed for 30 min to 3 μM of silver ions before and after 5 min of 10% v/v of tripan blue staining. Scaling bars: 20 μm.

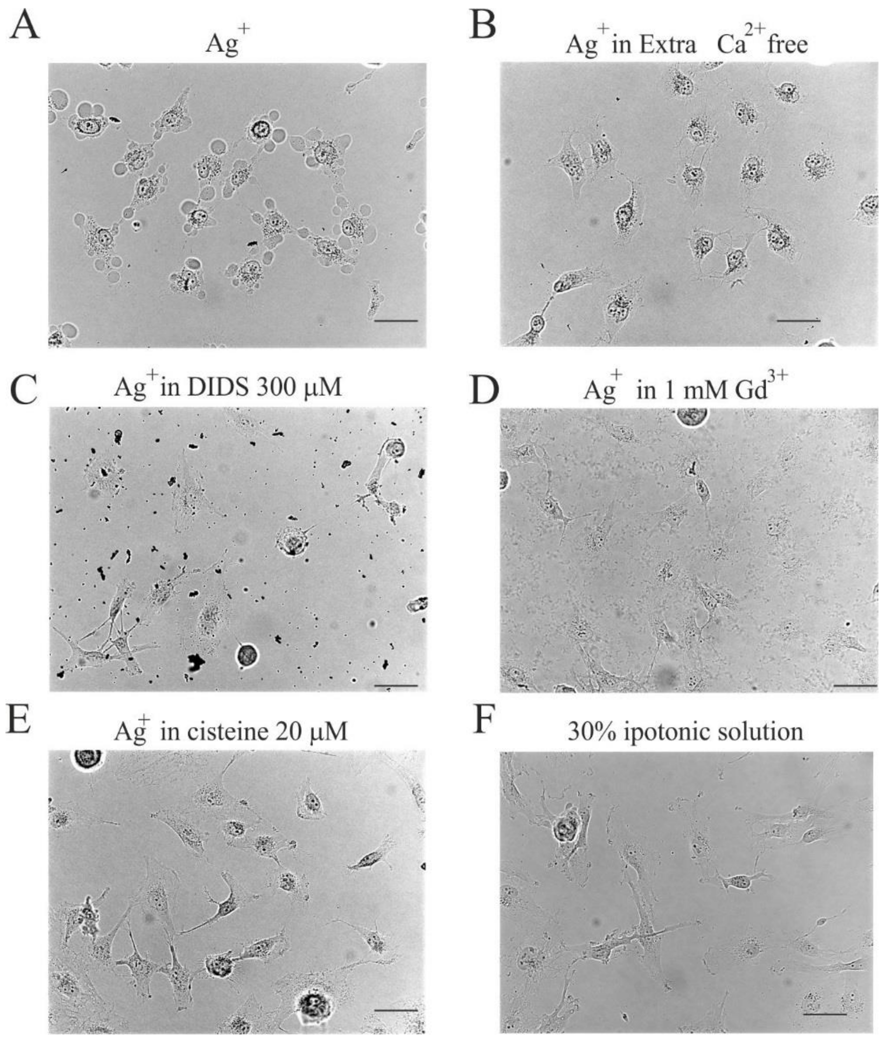

Supplementary Fig. S5. Blockers of IAg abolished silver ions induced morphological changes in U251 cells. A) Blebs formation after 30 minutes of 3 μM silver ions treatment in U251 cells. Blebs formation is abolished when Silver ions is applied in Ca^{2+}-free medium (**B**) and when silver ions was co-applied with 300 μM DIDS (**C**), 1 mM Gd^{3+} (**D**) or 20 μM cysteine (**E**). Ipotonic conditions do not allow blebs formation, although swelling was detect (**F**). Scaling bars: 20 μm.
